# The Effect of Coenzyme Q10/Collagen Hydrogel on Bone Regeneration in Extraction Socket Prior to Implant Placement in Type II Diabetic Patients: A Randomized Controlled Clinical Trial

**DOI:** 10.3390/jcm11113059

**Published:** 2022-05-29

**Authors:** Mostafa Ghanem, Lamia Heikal, Hagar Abdel Fattah, Adham El Ashwah, Riham Fliefel

**Affiliations:** 1 Department of Oral and Maxillofacial Surgery, Faculty of Dentistry, Alexandria University, Champollion Street, Alexandria 21526, Egypt; mostafaghanem333@gmail.com (M.G.); adhm.elashwah@alexu.edu.eg (A.E.A.); 2 Department of Pharmaceutics, Faculty of Pharmacy, Alexandria University, Champollion Street, Alexandria 21526, Egypt; lamia.heikal@alexu.edu.eg; 3 Department of Oral Biology, Faculty of Dentistry, Alexandria University, Champollion Street, Alexandria 21526, Egypt; hagar.sherif@alexu.edu.eg; 4 Experimental Surgery and Regenerative Medicine (ExperiMed), Ludwig-Maximilians University (LMU), Fraunhoferstrasse 20, 82152 Planegg-Martinsried, Germany; 5 Department of Oral and Maxillofacial Surgery and Facial Plastic Surgery, Ludwig Maximilians University, Lindwurmstrasse 2a, 80337 Munich, Germany

**Keywords:** CoQ10, collagen, bone regeneration, extraction, tooth, implant, diabetes

## Abstract

Background: The healing of an extraction socket leads to alveolar ridge resorption that can hinder future implant placement and further rehabilitation with special concerns in diabetes mellitus. Coenzyme Q10 (CoQ10) has been developed as a new material for alveolar socket augmentation. The aim of this study was to investigate the effect of CoQ10 hydrogel on bone regeneration after extraction of mandibular teeth in Type II diabetic patients. Methods: This trial was registered under the number NCT05122299 and included eighteen patients. The hydrogel was first prepared and characterized. After tooth extraction, the hydrogel was placed in the extraction sockets. Bone formation was evaluated three months after tooth extraction. Results: The bone density was significantly higher in the CoQ10 group than the other two groups measured on cone beam computed tomography (CBCT). The relative gene expression of Runt-related transcription factor 2 (RUNX2) and Osteopontin (OPN) showed significant increase in the presence of CoQ10. Histomorphometry revealed significantly less fibrous tissue in the CoQ10 group in comparison to the control or collagen group. Conclusion: The local application of CoQ10 after tooth extraction provided a simple, inexpensive, yet effective treatment facilitating bone formation and healing in the extraction sockets of diabetic patients.

## 1. Introduction

Tooth extraction is the most common and routine dental procedure performed due to traumatic dental injury, progressive periodontal disease, or endodontic lesions [1]. One of the most important challenges after tooth extraction is the preservation of the residual alveolar ridge for future placement of the dental implants and further rehabilitation [2].

The healing of the extraction socket leads to horizontal and vertical alveolar ridge resorption that can make implant placement difficult or affect the functional and aesthetic outcomes [3]. Multiple factors play role in bone resorption after tooth extraction including the number of teeth extracted, bone density, infection, or hormonal dysregulation including that associated with diabetes mellitus [4]. 

Diabetes is a common metabolic disorder characterized by chronic hyperglycemia, an inability to regulate blood glucose due to insulin deficiency or resistance that produces an inflammatory effect, resulting in decrease in bone formation [5], and leading to a deteriorated socket condition with an extended healing period [6], inhibition of osteoblastic differentiation, impairment of parathyroid hormone activity which regulates phosphorus, and calcium metabolisms [5]. All these problems affect osseointegration and long-term success of dental implants. Therefore, preservation of alveolar bone dimensions following tooth extraction is advantageous [7]. 

Alveolar socket preservation (ASP), also known as socket augmentation, is a procedure in which biomaterials are placed in the socket of the extracted tooth at the time of extraction to minimize dimensional changes of hard and soft tissue after tooth loss [8]. Numerous different methodologies and materials have been used, such as autograft, allograft, xenograft, and alloplastic materials. However, they have the disadvantages of defect size limitation, increased surgical time, and donor site morbidity [9]. 

To overcome their drawbacks, new materials have been developed more recently including but not limited to ubiquinone, also known as Coenzyme Q10 (CoQ10) [10], which is a lipid-soluble vitamin-like compound present in the inner membrane of the mitochondria of every cell of the body [11]. The structure of CoQ10 consists of a benzoquinone ring and a lipophilic isoprenoid side chain with ten isoprenyl units in the case of humans, which determines its low polarity and allows its fast diffusion through mitochondrial membrane [12]. Recently, CoQ10 has gained attention for its therapeutic application for several disorders including cardiovascular diseases [13], inflammation [14], human fertility [15], and diabetes mellitus [16]

In diabetes mellitus, CoQ10 helps to control blood glucose level and decrease glycosylated hemoglobin [17]. It also plays an important role in the protection of the cells from damage [18], and promotes osteoblast proliferation and differentiation [19]. It also plays an important role in treatment of periodontal diseases [20].

CoQ10 is a potent molecule, but its high molecular weight and low aqueous solubility hinder its use as a therapeutic agent [21]. To avoid these obstacles, various topical drug delivery systems have been explored and developed to overcome these limitations [22].

Collagen is a natural biodegradable and bioresorbable material, which has been used in a wide range of drug delivery systems due to the ease of extraction into an aqueous solution and molded into various forms [23]. As a natural material of low immunogenicity, it exhibits an extremely high biocompatibility with low antigenicity. It is a versatile material whose degree of crosslinking can be manipulated to control the rate of drug release [24].

Although collagen has been used extensively, no studies have evaluated the combination of collagen with CoQ10 in bone regeneration and healing of soft tissue. Thus, the aim of this study was to (a) evaluate the physicochemical properties of the CoQ10/Collagen hydrogel; (b) investigate the effect of CoQ10/collagen hydrogel on bone regeneration after extraction of mandibular teeth in Type II diabetic patients based on radiographic and histologic evaluation; and (c) explore the bone markers expressed during socket healing.

## 2. Materials and Methods

### 2.1. Ethical Approval and Registration

The present study was a randomized controlled single-blind, single-center clinical trial conducted at the Department of Oral and Maxillofacial Surgery (Faculty of Dentistry, Alexandria University) between November 2020 to October 2021. 

The study protocol was reviewed and approved by the institutional review board of the Faculty of Dentistry, Alexandria University (IRB No. 001056–IORG 0008839-0183-10/2020) and performed in accordance with the Declaration of Helsinki on experimentation involving human subjects. This trial was registered at (https://clinicaltrials.gov/, accessed on 10 April 2022) under the protocol identifier: NCT05122299 and reported according to the Consolidated Standard of Reporting Trials (CONSORT) guidelines [25].

### 2.2. Sample Size Calculation

An a priori power analysis was performed with G*Power Version 3.1.0 software [26] and suggested a sample size of 8 patients per group for a power of 80% with a 95% confidence level. Sample size was calculated to be 5 patients per group, increased to 6 to make up for lost to follow up with total sample size of 18 for the three groups. Sample size was based on Rosner’s method [27] calculated by Brant’s sample size calculator at the University of British Columbia (https://www.stat.ubc.ca/~rollin/stats/ssize/n2.html, accessed on 10 April 2022).

### 2.3. Study Participants

Eighteen patients in need of preservation of the extraction socket after the extraction of teeth planning for further prosthetic rehabilitation were enrolled in this study. All patients were informed about the nature of the study and gave their written consent.

### 2.4. Inclusion and Exclusion Criteria

The patients were included in the study after fulfilling the following inclusion criteria: (a) age range between 30–60 years with no gender predilection; (b) single or multiple mandibular teeth requiring extraction; (c) controlled Type II diabetic patients with glycosylated hemoglobin (HbA1C) levels less than 7; (d) good oral hygiene; and (e) no history of bruxism/parafunctional habits. The patients were excluded on the following criteria: (a) having uncontrolled severe systemic illness other than diabetes that may contraindicate the surgery; (b) smokers; (c) suffering from osteoporosis or hypersensitivity.; or (d) suspected allergy to any ingredients of the tested material.

### 2.5. Randomization and Allocation Concealment

The patients were randomly allocated into three groups (n = 6 per group). Control (ungrafted extraction sockets): no intervention after the tooth extraction. Collagen (Positive control): application of collagen hydrogel only in the extraction socket. Coenzyme Q10 (Test group): Application of CoQ10/Collagen hydrogel in the extraction socket. All the patients received dental implants at the site of grafting three months later. Randomization was performed using an online service (https://www.randomizer.org/, accessed on 10 April 2022) [28]. The Collagen and CoQ1o/Collagen groups had the same surgical procedures. 

After obtaining informed consent, the participants were randomly allocated using sequentially numbered, opaque, sealed, and stapled envelopes (SNOSE). Immediately after tooth extraction, the envelope was opened by the maxillofacial surgeon to determine the assigned treatment group. 

### 2.6. Preparation of the CoQ10/Collagen Thermoresponsive Hydrogel

The CoQ10/Collagen hydrogel was prepared by first dissolving 2 g of collagen in 5 mL of 0.1 M acetic acid solution to reach a stock collagen solution of 200 mg/mL concentration. The solution was then sonicated for 30 min to remove air bubble. Beta-Glycerophosphate (10% *w*/*w*, G9422, Sigma Aldrich, Taufkirchen, Germany) was then added drop by drop to the collagen solution until pH 7.4 was attained. 

For preparation of CoQ10/ collagen loaded hydrogel, 150 mg/mL CoQ10 was dispersed in a 25% *w*/*w* poloxamer 407 solution and kept at 4 °C. Collagen solution was then added to reach a final concentration of 100 mg/mL gel solution. Poloxamer 407 with collagen but without CoQ10 served as the positive control.

### 2.7. Evaluation of Gelation Behaviour 

The gelation behavior of the CoQ10/collagen and unloaded thermoresponsive hydrogels were assessed by measuring gelation time at different temperatures (4 °C, 25 °C, and 37 °C). Gelation time was evaluated by vial tilting method [29]. Both hydrogels were prepared at fixed polymer concentration (poloxamer 407, 16758, Sigma Aldrich, Taufkirchen, Germany) (25% *w*/*w*) and fixed collagen concentration (200 mg/mL). The gelation time was considered at the point when there was no flow for more than 1 min after repeatedly inverting the vial with 1 mL mixture of the collagen or the CoQ10/Collagen solution. The experiments were performed in triplicates.

### 2.8. In-Vitro Release of CoQ10 

In vitro release of CoQ10 from the prepared hydrogel was performed using the dialysis method. Briefly, presoaked dialysis bags (Visking^®^ 36/32, 24 mm, MWCO 12,000–14,000, Serva, Wichita Falls, TX, USA) were filled with either 1 mL of CoQ10/Collagen hydrogel, collagen hydrogel or CoQ10 free suspension. The tubes were then placed in 60 mL release medium (PBS; Phosphate buffer saline) to ensure sink condition at 100 rpm and 37 °C. At pre-determined time intervals (0.25, 0.5, 1, 2, 4, 6, and 24 h), aliquots of sample were withdrawn and replaced with equal amounts of fresh PBS buffer. The amounts of CoQ10 released into each PBS aliquot was quantified spectrophotometrically at λ_max_ = 273 nm. The cumulative % CoQ10 released was calculated and corrected relative to the initial drug content. The study was performed in triplicate and the results were expressed as Mean ± SD.

### 2.9. Swelling Ratio (SR) 

The swelling ratio of collagen and CoQ10 loaded collagen was measured by immersing the hydrogel in deionized water at room temperature at (0.5, 1, 6, and 24 h). At the end of each incubation period, the hydrogels were washed with deionized water and blot-dried using filter paper. After carefully blotting the water from the surface, the weights of the swollen hydrogel were measured with a standard laboratory balance to measure the wet weight. The dry weight is the weight of the sample before immersing in deionized water. The swelling ratio (SR) was determined using the following equation [30]:

(1) SR=[(Wwet−Wdry)/Wdry]

where Wwet is the final mass of the hydrogels after swelling in deionized water and Wdry is the initial mass of the hydrogel sample. All experiments were carried out in triplicates.

### 2.10. Pre-Surgical Procedures 

Before surgery, each patient underwent initial scaling and root planning with instructions for maintaining good oral hygiene. Demographic data were taken for each patient in addition to a detailed medical and dental history. The patients were first examined clinically by extraoral examination for assessment of any facial asymmetry and the presence of palpable lymph nodes and an intraoral examination by palpation of the entire oral and para-oral tissues and then examination of the teeth to be extracted. As for radiographic examination, orthopantomograms (OPG) were taken for all the patients to detect any lesions related to teeth to be extracted or approximation to the inferior alveolar canal while cone beam computed tomography (CBCT) was taken immediately after extraction (T0) and three months post-operative prior to implant insertion (T1).

### 2.11. Extraction of Teeth 

The teeth extraction was carried out following the institution’s guidelines for the management of diabetic patients. Prophylactic antibiotic (1 gm of amoxicillin/clavulanic acid, Augmentin, GlaxoSmithKline, Middlesex, UK) was administrated one hour before the surgery and the patients were instructed to rinse the mouth with Chlorohexidine HCl mouth wash (Hexitol, The Arab Drug Company, Cairo, Egypt) for 30 s.

**Figure 1:**
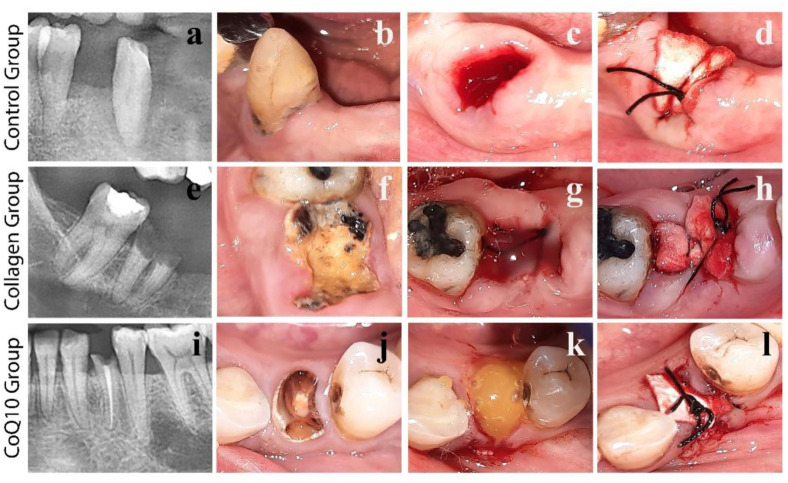
Augmentation of dental sockets after tooth extraction: (**a**) pre-operative OPG showing the tooth to be extracted in the control group; (**b**) mandibular right canine indicated for extraction due to periodontal disease; (**c**) fresh extraction socket left ungrafted after tooth extraction; (**d**) surgical suture after application of a collagen membrane; (**e**) pre-operative OPG showing the tooth to be extracted in the collagen group; (**f**) badly destructed mandibular right second molar indicated for extraction; (**g**) fresh extraction socket filled with collagen hydrogel only after tooth extraction; (**h**) surgical suture after application of a collagen membrane on top of the collagen hydrogel; (**i**) pre-operative OPG showing the tooth to be extracted in the CoQ10 group; (**j**) badly destructed mandibular left first premolar indicated for extraction; (**k**) fresh extraction socket filled with CoQ10/Collagen hydrogel after tooth extraction; and (**l**) surgical suture after application of a collagen membrane on top of the CoQ10/Collagen hydrogel.

For all groups, the surgical procedures were carried out by the same oral and maxillofacial surgeon (MG) under local anesthesia (Articaine HCL with epinephrine 1:100,000, 3M ESPE, Seefeld, Germany) to minimize operator-dependent variables. The teeth were extracted in atraumatic flapless manner with rotation and traction movements, using dental forceps and elevators with minimal soft tissue reflection and without causing any trauma to the underlying alveolar bone. The socket was then gently irrigated with normal saline and hemostasis was achieved by digital mechanical pressure. The socket was then augmented starting from the most apical area to the most crestal area of the socket with collagen in the positive control, CoQ10/Collagen hydrogel in the study group or the socket was left ungrafted in the negative control. After placing the grafting material, a collagen membrane (RD2502, PARASORB RESODONT^®^, RESORBA Medical GmbH, Nürnberg, Germany) was placed and secured in place by 3/0 silk sutures (Ghatwary Medical Supply; GMS, Alexandria, Egypt) to confirm socket closure (Figure 1). The sutures were removed after 1 week. 

### 2.12. Post-Operative Care

All patients received the same antibiotic twice daily as well as non-steroidal anti-inflammatory (Diclac-ID 150 mg, Diclofenac Sodium, Hexal/MinaPharm, Cairo, Egypt) every eight hours for five days. Early follow-up was performed immediately at the first week after graft placement to detect any pain or infection. All patients were followed-up until full healing had occurred. 

### 2.13. Pain and Post-Extraction Complication Assessment

Early follow-up was performed immediately at the first week after graft placement to detect any pain according to Mankowski pain scale (Appendix A) or infection. Post-operative pain was recorded through telephone interviews which are on the evening of the day of tooth extraction and then daily for 6 days after tooth extraction and finally with one personal interview on the 7th day at the clinic. During each interview, the patients were asked about their pain intensity using a scale of 0–10, with no pain marked as 0 and intolerable pain marked as 10. In addition, the patients were asked about any possible complications, such as swelling or bleeding.

### 2.14. Cone Beam Computed Tomography (CBCT) Acquisition

CBCTs were acquired using J. Morita R100 (J. MORITA MFG. CORP., Kyoto, Japan). The scans were performed with a field of view (FOV) of W100 mm × H50 mm. The volume was reconstructed with 0.160 mm isometric voxel size. The tube voltage was 90 kVp and 8 mA and an exposure time of 20 s. The data were exported as DICOM (Digital Imaging, and Communications in Medicine) files.

### 2.15. Radiographic Evaluation 

To assess bone regeneration in the extraction socket, CBCTs were taken at two different timepoints: (a) immediately after tooth extraction serving as the baseline to measure the bone density in the extraction socket and height/width of the alveolar ridge (T0); and (b) after three months and before implant surgery for accurate positioning of the implants (T1). 

The density of the newly formed bone formed after three months inside the extraction sockets in the three different groups was compared to the baseline immediately after tooth extraction. The bone density was calculated by taking mean readings of the gray intensity measured by ImageJ software [31] (Version 1.53n, 7 November 2021, US National Institutes of Health, Bethesda, MD, USA, https://imagej.nih.gov/ij/, accessed on 10 April 2022). The Mature bone tissue was defined as high intensity areas (HIA), while fibrin matrix was defined as the weak intensity areas (WIA). At the demarcated region of interest (ROI) which is the dental socket area, the pixels of light gray tones corresponding to areas of bone formation were selected to calculate the bone density. The percentage of formed bone inside the extraction socket was calculated according to the following formula [32]:

(2) $$\mathrm{Percentage}\;\mathrm{of}\;\mathrm{bone}\;\mathrm{formed}\;\mathrm{in}\;\mathrm{extraction}\;\mathrm{socket}\;(\%)=\frac{b-a}{x}\;\;\text{×}\;100$$

where x = mean gray value of the normal bone adjacent to the extraction socket; a = mean gray value of bone density in the socket immediately after extraction; and b = mean gray value of bone density in the socket at three months after extraction.

As for the analysis of the alveolar bone height and width, the measurements were performed on the CBCTs according to Jung et al. [33]. The radiographic measurements taken at (T0) and (T1) were superimposed using the original DICOM data and processed using Blue Sky Plan program (version 2.19, Blue Sky Bio, LLC, Grayslake, IL, USA, https://blueskybio.com/home, accessed on 10 April 2022). A computer-assisted superimposition was done in the selected areas using stable landmarks including the anterior nasal spine, genial tubercle, mental foramen, and inferior alveolar nerve canal, where no changes had taken place during the last three months. The two data sets were aligned, manually checked for perfect match, and the measurements were made using the same reference points and lines. 

To set a reference, the most apical point of the extraction socket was defined in the baseline image and two reference lines were subsequently drawn. The vertical reference line was drawn in the center of the extraction socket crossing the apical reference point. The horizontal reference line was drawn perpendicular to the vertical line crossing the apical reference point. Then, the measurements with respect to these reference points and lines were then performed in the center of the extraction socket, which are: (a) horizontally, change in the ridge width measured in millimeters at three different levels (1 mm, 3 mm, 5 mm) below the most coronal aspect of the crest (HW-1, HW-3, HW-5); and (b) vertically, changes in ridge height measured in millimeters at the buccal and lingual aspect. The bony changes were confirmed by subtracting the values at three months (T1) from the values at the baseline (T0)**.**

The volume of the extraction sockets at T0 and T1 was also evaluated according to Anitua et al. [34]. For measuring the volume, the CBCT scans at T0 and T1 were imported into the ImageJ software and the readings were recorded. In order to calculate the defect volume, extraction socket was considered as a cone and the volume of the socket was determined according to the following equation:

(3) V=13 πr2h

where V is the volume of the extraction socket, r is half of the most coronal width of the socket, and h is the apicocoronal distance of the middle of the socket. 

However, when an inter-radicular septum was present, the total volume of the extraction socket was the sum of the volumes of the mesial root and the distal root. The new socket volume was calculated on the CBCT scan obtained three months after surgery.

### 2.16. Presurgical Planning for Implant Site

**Figure 2:**
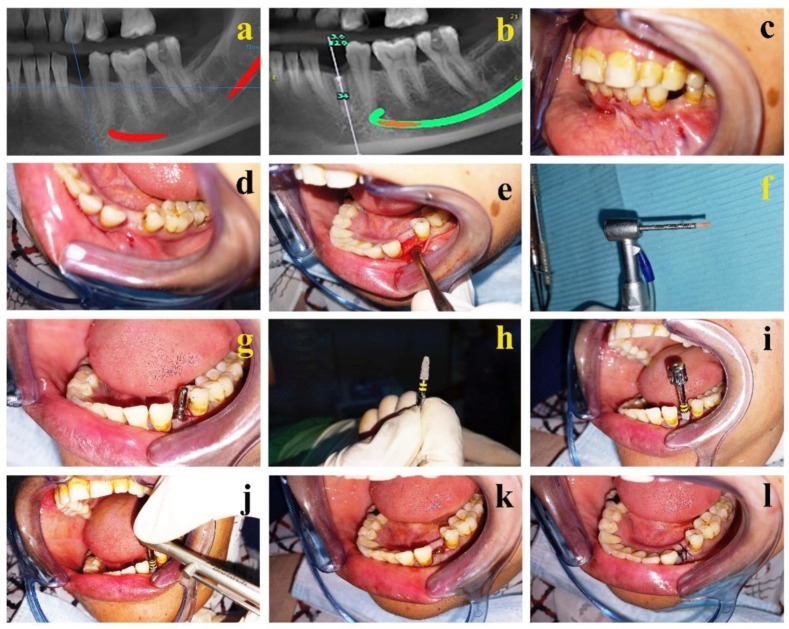
Surgical re-entry at 3 months for bone core biopsy and implant placement: (**a**) pre-operative CBCT of the extraction site at 3 months showing complete healing of the extraction socket; (**b**) pre-operative CBCT of the virtual implant placed at the extraction site; (**c**) pre-operative clinical presentation of the inter-occlusal space in the centric occlusion before the implant placement; (**d**) intra-oral picture showing complete mucosal healing after extraction of the first premolar before the implant placement; (**e**) reflection of a full mucoperiosteal flap; (**f**) bone core biopsy obtained harvested from the augmented area by the trephine bur before implant site preparation; (**g**) parallel pin in place showing the parallelism of the implant to the neighboring teeth; (**h**) macrostructure of the NeoBiotech tapered resorbable blast media (RBM) dental implant (3.5 × 11.5 mm); (**i**) implant inserted into the osteotomy site; (**j**) complete insertion of the implant in the osteotomy site using manual torque wrench; (**k**) cover screw placed over the dental implant before closure of the flap; and (**l**) mucoperiosteal flap repositioned and sutured.

Before the implant surgery, CBCT of all the examined ridges were acquired to evaluate the healing of the extraction socket, the amount of augmented bone, assess the labiolingual width and estimate the size of the implant to be inserted (Figure 2a). Virtual dental implant (3.6 mm in diameter cylinder with a flat-end top) was placed in the OnDemand dental planning software (OnDemand3D Technology, Tustin, CA, USA) (Figure 2b).

### 2.17. Bone Collection during Implant Surgery 

Briefly, a mucoperiosteal flap was raised to expose the alveolar ridge. No releasing incisions were performed to preserve the periosteum and ensure adequate vascularization and venous drainage in the operative zone. Initially, a core biopsy of 1.7 mm internal diameter and a depth of 8–10 mm was performed from the alveolar site to obtain bone samples from the implant beds using a trephine bur (Inside shaft ø1.7 mm, 2.35 mm × 30 mm length; 7 teeth graduation, Helmut Zepf, Seitingen-Oberflacht, Germany) (Figure 2a–f). The obtained bone samples were divided into two pieces where one piece was stored at −80 °C for RNA isolation and the other piece was fixed in 10% neutral buffered formalin (HT501128, Sigma-Aldrich, Taufkirchen, Germany) for histological investigations.

### 2.18. Implant Placement Surgery

Re-entry of the socket was performed after three months for implant placement. All the patients received prophylactic antibiotic (1 gm of amoxicillin/clavulanic acid, Augmentin, GlaxoSmithKline, Middlesex, UK) one hour before the surgery and continued for 7 days postoperatively. The patients were instructed to rinse the mouth with Chlorohexidine HCl mouth wash (Hexitol, The Arab Drug Company, Cairo, Egypt) for 30 s. All patients were instructed to administrate non-steroidal anti-inflammatory (Diclac-ID 150 mg, Diclofenac Sodium, Hexal/MinaPharm, Cairo, Egypt) if needed. 

After obtaining the core biopsy, additional osteotomy was performed at the implant site according to the standard technique for implant placement, and the implants (IS-II Active; Neobiotech Co., Ltd., Seoul, Korea) were inserted at a speed of 800 rpm and torque of 45 N-cm in each surgical site. Root form endosteal implant with diameter ranging from 3.6 mm to 5.5 mm was placed depending on the mesiodistal and the buccolingual bone thickness. The surface of these implants is sandblasted and acid-etched. Following copious irrigation with physiological saline solution, the mucoperiosteal flap was repositioned and sutured with 3/0 non-resorbable sutures (Ghatwary Medical GMS, Alexandria, Egypt) (Figure 2g–l). The sutures were removed one week postoperatively. 

### 2.19. Measurement of Implant Stability

Resonance frequency analysis (RFA) measurements of implant stability were performed via Osstell ISQ (Osstell AB, Gothenburg, Sweden), which expresses the stability of implant as implant stability quotient (ISQ). The implant stability measurement was examined at the time of insertion of the implants (primary stability) and at loading, three months later, for the three groups following the manufacturer’s instructions. A Smart Peg resonator was attached to the implant and each implant was measured in two different directions mesiodistally and buccolingually. The measurements from both directions were calculated as the arithmetic mean. The value obtained by the device was shown as an ISQ value. As suggested by the manufacturer, an Implant Stability Quotient (ISQ) ≥70 represented high level of implant stability while an ISQ <60 represented low level of implant stability.

### 2.20. RNA Isolation and Pooled RNA Quantification

All the bone samples were handled following a Standard Operation Procedure (SOP) defined at the beginning of the study. Total RNA was obtained from bone core biopsy at the implant site at the day of implant surgery using BIOZOL reagent (BSC51M1, BioFlux, Beijing, China) according to the manufacturer’s instructions. The bone samples from each group were pooled together to provide sufficient RNA yield for analysis and minimize individual patient differences. Briefly, the obtained bone was placed in the homogenization tubes containing 500 µL of pre-chilled BIOZOL Reagent and homogenized by the disperser (IKA ULTRA-TURRAX^®^ T25 digital, IKA-Werke GmbH & Co. KG, Staufen, Germany) for a total of 45–60 s at a speed of 18,000 rpm for 20 s at room temperature, 3 times to disrupt the bone. After homogenization, the samples were centrifuged for 15 min at 10,000 rpm at 4 °C to separate the lysate from the bone debris. Following centrifugation, the clear supernatant was pipetted up into a new safe locked microcentrifuge tube. Then, 100 µL Chloroform (438613, CARLO ERBA Reagents GmbH, Emmendingen, Germany) was added and the samples were vortexed again prior to centrifugation for 15 min using the microcentrifuge (Sigma 1–14k, Sigma Laborzentrifugen GmbH, Osterode am Harz, Germany) at 12,000× *g* for 15 min at 4 °C. The aqueous phase was transferred to a new tube, and an equal volume of ice-chilled propan-2-ol (415158, CARLO ERBA Reagents GmbH, Emmendingen, Germany) was added, mixed gently and left overnight at 4 °C to allow RNA precipitation. Samples were then centrifuged at 12,000× *g* for 30 min at 4 °C for pellet collection. The propan-2-ol was discarded, and the pellet was washed with 1 mL of 75% (*v*/*v*) absolute ethanol (E/0650DF/17, Fisher Scientific, Loughborough, UK) before centrifugation at 7500× *g* for 15 min at 4 °C. The ethanol was then discarded, and the pellet was left to air dry for 1 h. The dry pellet was then dissolved in 30 μL of nuclease-free water (B1500S, New England Biolabs, Ipswich, MA, USA) before storage at −80 °C in an ultra-freezer. The concentration and quality of the total RNA extracted was then measured by the NanoDrop 1000 spectrophotometer (Thermo Scientific, Waltham, MA, USA). 

### 2.21. Quantitative Reverse Transcription Polymerase Chain Reaction (RT-qPCR) of Osteogenic Genes 

The RT-qPCR was carried out in QuantStudio 1 Real-Time PCR System (Applied Biosystems; Thermo Scientific, Waltham, MA, USA) using one-step HERA SYBR^®^ Green RT-qPCR (WF10303001, Willowfort, Birmingham, UK) to analyze the expression of the osteogenic genes according to the manufacturers’ instructions. 

Briefly, for one-step, a master mix was prepared using 1 μL RT Enzyme Mix (20×, 10 μL HERA SYBR^®^ Green RT-qPCR Master Mix (2×), 1 μL Forward primers 20× (200 nM), 1 μL Reverse primers 20× (200 nM), 6.4 μL Nuclease-free water, and 0.6 μL RNA template (up to 250 ng). A reaction solution of 20 µL per sample was dispensed in each tube. The RT-qPCR step involved reverse transcription at 55 °C for 15 min. The PCR reaction system was performed according to protocols provided with the qPCR detection kit. The PCR reaction conditions included enzyme activation at 95 °C for 5 min followed by denaturation at 40 cycles of 95 °C for 10 s, then annealing 60 °C for 30 s, and eventually the melting curve was analyzed.

Glyceraldehyde-3-phosphate (GAPDH) served as the housekeeping gene and was determined using the comparative cycle threshold (CT) method to calculate 2^−ΔΔCt^. First, (∆CT) was calculated as the difference between the CT mean of each target gene and that of the endogenous control gene (GAPDH). Then, ∆∆CT was calculated as the differences of the ∆CT values of the calibrator samples and the ∆CT values of the test samples. Finally, the fold change, or the mRNA expression level, was calculated using the formula: 2^−ΔΔCt^ to quantify the relative gene expression. The ungrafted sockets served as negative control and extraction sockets filled with collagen only served as the positive control. The results were analyzed using SDS 2.0 software (Life Technologies, Grand Island, New York, NY, USA) and presented as the relative mRNA expression level. 

The primer sequences were listed as follows: **Glyceraldehyde-3-phosphate (GAPDH)**, forward: 5′-CAACTACATGGTTTACATGTTC-3′ and reverse: 5′-GCCAGTGGACTCCACGAC-3′, **Collagen type I alpha 1 chain (COL1A1)**, forward: 5′-AGGGCTCCAACGAGATCGAGATCCG-3′ and reverse: 5′-TACAGGAAGCAGACAGGGCCAACGTCG-3′, **Runt-related transcription factor 2 (RUNX2)**, forward: 5′-TCTTCACAAATCCTCCCC-3′ and reverse: 5′-TGGATTAAAAGGACTTGGTG-3′, **Osteoclacin (OCN)**, forward: 5′-GGCACAAAGAAGCCGTACTC-3′ and reverse: 5′-CACTGGGCAGACAGTCAGAA-3′, **Osteopontin (OPN)**, forward: 5′-CTGATGAACTGGTCACTGATTTTC and reverse: 5′-CCGCTTATATAATCTGGACTGCTT-3′.

### 2.22. Histological Examination

Overall, 18 fixed cylindrical bone cores were first decalcified in 5% TCA (Trichloroacetic acid) for 3 days followed by dehydration in a serial concentration of ethanol after being rinsed with water. The biopsies were cleared in xylene, infiltrated and embedded in paraffin wax. Thin sections of 4–5 μm were cut using the microtome (KD-2258, Zhejiang Jinhua Kedi Instrumental Equipment, Jinhua, China). All specimens were stained with Hematoxylin and Eosin (H&E) and Gomori Trichrome staining and observed by the light microscope (Optika, B-290 series, Ponteranica, Italy) to evaluate the type and quality of the formed organic matrix and bone. Photomicrographs were taken using a digital camera (Optika, C-B10, Ponteranica, Italy) at 40, 100 and 400× magnification and the images were saved on a computer. 

### 2.23. Histomorphometric Analysis

Computer-assisted histomorphometry analysis was performed for the H&E stained sections to compare between the mean percent of the newly formed bone in the three groups using the ImageJ software. The specimens were blindly evaluated histomorphometrically by a single examiner. Sections at standardized magnification of 100× were evaluated from the most coronal to most apical extent under light microscopy. The following parameters were measured: percentage of bone formed and percentage of fibrous tissue per field. 

The ratio of new bone to total area was calculated using the following equation:

(4) $$\mathrm{Percentage}\;\mathrm{of}\;\mathrm{new}\;\mathrm{bone}\;\mathrm{area}=\frac{\mathrm{New}\;\mathrm{Bone}\;\mathrm{Area}}{\mathrm{Total}\;\mathrm{Area}}\times 100$$

### 2.24. Primary and Secondary Outcomes

Primary outcomes: The mean bone density, the dimensional changes in the width and height of the extraction socket, and the socket volume immediately after extraction and 3 months after the procedure using CBCT. The expression of bone markers during healing of the extraction socket using RT-qPCR and, finally, the quality of bone formed in the histological sections.

Secondary outcomes: Postoperative pain following the extraction of the teeth assessed for 7 days using Mankowski Pain Scale.

### 2.25. Statistical Analysis

A normality test was used to evaluate the data using descriptive statistics as well as the Shapiro–Wilk and box plots to evaluate the distribution of the data. All the variables were not normally distributed. Data was presented using mainly Median, Inter Quartile Range (IQR) in addition to Mean, Standard deviation (SD) and 95% Confidence Interval (CI). The level of significance was indicated at * *p* ≤ 0.05, ** (*p* < 0.01) or *** (*p* < 0.001). Groups were compared using Kruskal–Wallis test followed by pairwise comparisons when results were significant and *p* values were adjusted by Bonferroni Correction. Differences in pain scores and bone width across three time intervals were assessed using Friedman Test and followed by pairwise comparisons while differences in bone density, volume and ISQ values immediately and after 3 months were assessed using Wilcoxon Sign Rank Test. All tests were two tailed. Data were organized in the Statistical Package for Social Sciences (SPSS) version 23.0 Statistics (IBM Corp., Armonk, NY, USA). All the graphs were performed using GraphPad Prism software version 9.3.1 for Windows (GraphPad Software, San Diego, CA, USA, https://www.graphpad.com/, accessed on 10 April 2022).

## 3. Results

### 3.1. Evaluation of Gelation Behavior

The gelation time was dependent on the temperature to which CoQ10/Collagen and Collagen hydrogels were exposed. Neither thermoresponsive hydrogels produced any hydrogel at 4 °C. At room temperature (25 °C); the gelation time of CoQ10/Collagen and Collagen hydrogels were 120 and 105 s, respectively. The gelation time was reduced from 120 to 45 s and from 105 to 30 s for CoQ10/Collagen and Collagen hydrogels when exposed to 37 °C.

### 3.2. In-Vitro Release of CoQ10 

**Figure 3:**
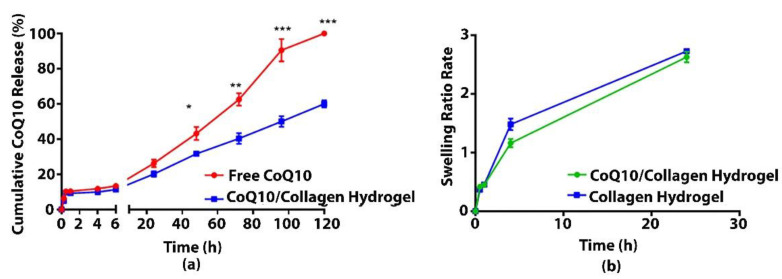
(**a**) In-vitro release profile of CoQ10 from CoQ10/Collagen hydrogel and from free CoQ10 in PBS at 37 °C, 100 rpm ensuring sink condition; (**b**) Swelling ratio as a function of time for CoQ10/Collagen and Collagen thermoresponsive gels Statistical Significance is achieved where * (*p* ≤ 0.05), ** (*p* < 0.01), *** (*p* < 0.001). All experiments were performed in triplicates.

CoQ10 in collagen thermoresponsive hydrogel showed a sustained release profile compared to free CoQ10 where 60 ± 2.5% CoQ10 was released from the hydrogel over a period of 5 days (Figure 3a). 

### 3.3. Swelling Ratio

The swelling ratio as a function of time for CoQ10/Collagen and Collagen thermoresponsive hydrogels was presented in Figure 3b. It was noted that Collagen hydrogel has absorbed more water initially than the CoQ10/Collagen hydrogel. However, the incorporation of CoQ10 particles in the collagen had controlled the swelling ratio of the hydrogel. The swelling rate of the hydrogels later increased with time. The maximum swelling ratio was observed up to 24 h. It is estimated that increasing the time led to more swelling of the hydrogel.

## 4. Clinical Outcomes

**Figure 4:**
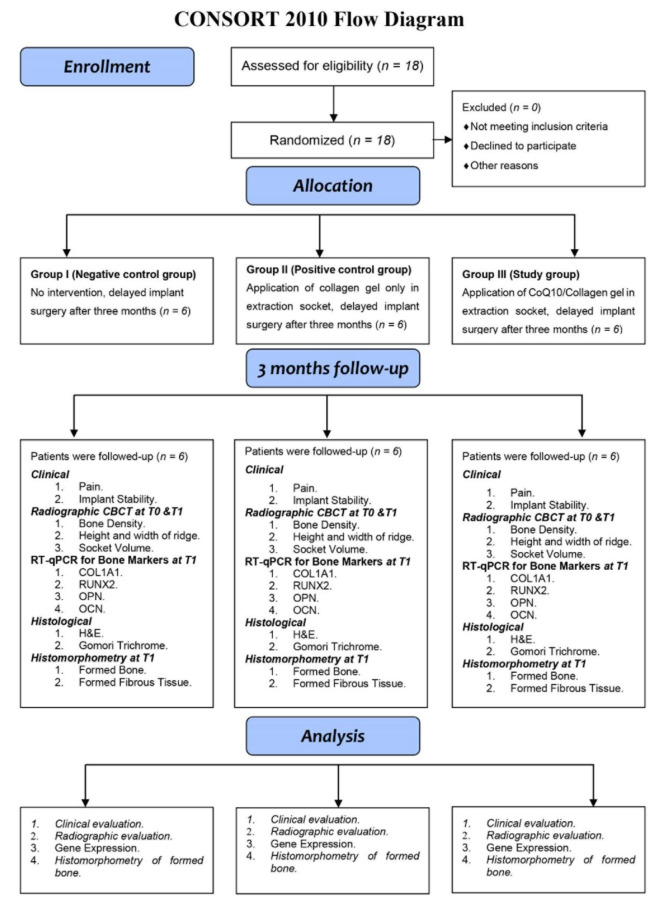
CONSORT 2010 Flow Diagram of the study.

This study included eighteen patients, 15 males and 3 females, with a mean age of 51.67 ± 6.28 years (ranging from 39 to 59 years) who completed the study and randomly allocated into either of the three groups. The patients have undergone simple dental extraction due to a periodontal disease, causing tooth mobility or the teeth being badly destructed The CONSORT flowchart of the randomized controlled clinical trial was presented in Figure 4.

### 4.1. Assessment of Post-Extraction Socket Healing and Pain

Clinical healing was free of infection or symptoms in all groups. No postoperative complications, such as dry socket or secondary infection, occurred. Pain at the extraction site was reported for seven days postoperatively.

Soft-tissue healing of the extraction sockets was uneventful and visually assessed as quick in the CoQ10 group. Almost complete soft-tissue closure was present 7 days after extraction in the CoQ10 group. At a similar timeframe, incomplete soft-tissue healing was observed in the other two groups. 

**Figure 5:**
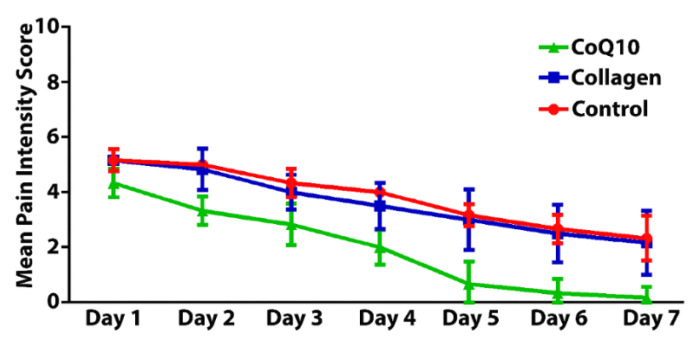
Frequency of pain intensity among the study groups at different time points during the post-extraction period.

The post-operative pain intensity was significantly higher in the control and collagen group than in the CoQ10 group (*p* ≤ 0.05). Although the pain intensity has decreased in the three groups from day 3 to day 7, CoQ10 reduced the postoperative pain scores significantly (*p* ≤ 0.05). Detailed description of the pain intensity was presented in Figure 5.

### 4.2. Implant Stability

The mean implant stability quotient (ISQ) at the insertion of the dental implants was recorded to be 61.8 ± 0.58, 56.6 ± 2.54, and 61.1 ± 0.89 ISQ for the ungrafted socket (Control), Collagen, and CoQ10 groups, respectively. At three months post-insertion, the ISQ decreased to 52.2 ± 0.74, 53.8 ± 2.17, and 56.6 ± 2.39 for the Control, Collagen, and CoQ10 groups, respectively (*p* = 0.304). There was statistically significant difference in the ISQ at the time of implant insertion and three months after insertion in each group (*p* = 0.028). However, there was no significant difference in the implant stability at insertion (*p* = 0.168) or at three months (*p* = 0.304) between the three different groups. Table 1 showed the implant stability at the insertion and three months after insertion.

### 4.3. Radiographic Outcomes 

The results of bone density, percentage of bone formation in the extraction socket, difference in the height and width of the alveolar socket, and percentage of volume reduction in the extraction sockets were summarized in Table 2. 

#### 4.3.1. Bone Density

The bone density represented as the mean gray value was not statistically significant between the control and collagen groups (*p* = 1.00). However, the bone density and percentage of bone formation in the CoQ10 group was significantly higher than the other two groups (*p* = 0.003).

#### 4.3.2. Bone Linear Outcomes

CBCTs of the extraction socket indicated that there was statistically significant less vertical bone loss on the buccal plate of bone in the CoQ10 compared to the collagen or ungrafted socket (Control) at three months (*p* = 0.011) with the most vertical loss in the ungrafted socket group. With respect to vertical bone loss on the lingual plate of bone, the CoQ10 group showed the least bone resorption to the other two groups, but the results were not statistically significant (*p* = 0.493). As for the horizontal bone loss as an indicator for the decrease in the socket width, there was decrease in the mean width of the alveolar socket in all the groups at 1 mm, 3 mm, and 5 mm. However, this reduction at the three levels was not statistically significant (*p* = 0.700, *p =* 0.644 and *p =* 0.262) respectively. 

#### 4.3.3. Bone Volume of the Extraction Socket

CBCT scan measured at the baseline (T0) and three months later (T1) revealed that there was a significant reduction of the socket volume between T0 and T1 (*p* = 0.028) in each group, while there was no significant difference in the volume of the socket in the three groups either immediately after extraction (*p* = 0.548) or at three months (*p* = 0.348).

### 4.4. Gene Expression Analysis 

**Figure 6:**
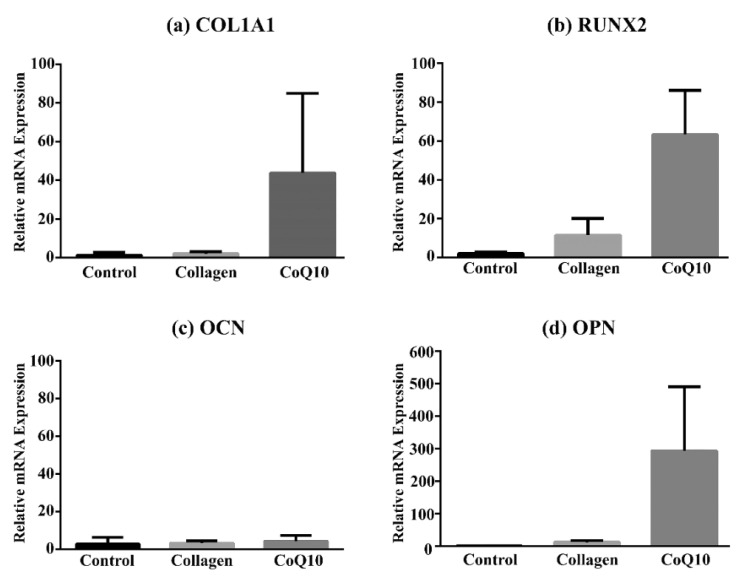
RT-qPCR of the mRNA expression of the selected bone formation markers, (**a**) collagen 1A1 (COL1A1), (**b**) Runt-related transcription factor 2 (RUNX2), (**c**) osteocalcin (OCN), and (**d**) osteopontin (OPN) at 3 months after healing of the extraction socket. Data are the representative of three independent experiments in the Control, Collagen and CoQ10 groups (*p* ≤ 0.05).

To understand the mechanism by which the bone healing is influenced in the presence of Collagen or CoQ10/Collagen hydrogel, RT-qPCR was performed and the bone formation-related genes were evaluated. A total of six bone samples in each group was analyzed. The expression of the selected genes at 3 months after healing of the extraction socket were shown in Figure 6. In CoQ10-treated sockets, the mRNA levels of COL1A1 (43.84 ± 41.20) were increased compared to non-treated sockets or sockets filled with collagen (1.41 ± 1.39 versus 2.10 ± 1.10, *p* = 0.051). Furthermore, mRNA expression of RUNX2 was significantly increased in CoQ10 group compared to the Control and Collagen group (2.09 ± 0.97, 11.58 ± 8.52, 63.38 ± 22.78, *p* = 0.027), respectively. The mRNA expression of OPN showed a significant increase in the presence of CoQ10, where a mean increase of 293.86 ± 197.24 in the CoQ10 group while only a mean of 1.62 ± 0.66 and 12.80 ± 5.48 were reported in the Control and Collagen group respectively (*p* = 0.027). The OCN did not show any significant difference in the mRNA expression in either of the three groups (*p* = 0.491).

### 4.5. Histological Analysis

All biopsies were successfully retrieved and processed for light microscopic examination and digitally labelled for histomorphometry. The histologic assessment of the specimens showed no evidence of marked inflammatory reactions or occurrence of foreign body reactions in any of the eighteen histological samples. 

In the control group, thin new spicules of immature woven bone were detected in various regions in the biopsy samples in addition to areas of thin trabeculae of spongy bone; the marrow tissue showed loose mesenchymal tissue with reduced cellularity and vascularity. Few inflammatory cells were also noted. Moreover, widened and empty osteocytes lacunae were observed. Dense fibrous tissue, comprising fibroblasts, collagen fibers, and small capillaries, was also observed in some specimens surrounding the sparse newly formed bone.

In the collagen group, immature spicules of woven bone and thin segments of trabecular bone were observed surrounded by moderate to thick fibrous tissue bundles that fill the bone marrow cavities. Numerous osteocytes lacunae were widened or empty. 

In the CoQ10/collagen group, Dense mature lamellar compact bone with primary and secondary osteons was revealed fused to the old bone. Typical trabecular bone structures were also observed with clearly distinguishable osteocytes, which is indicative of vital bone tissue. Resting and reversal lines were evident indicating active bone remodeling. Normal sized marrow cavities filled with dense mesenchymal tissue with mild fibrosis were also presented lined by flat endosteal cells. 

**Figure 7:**
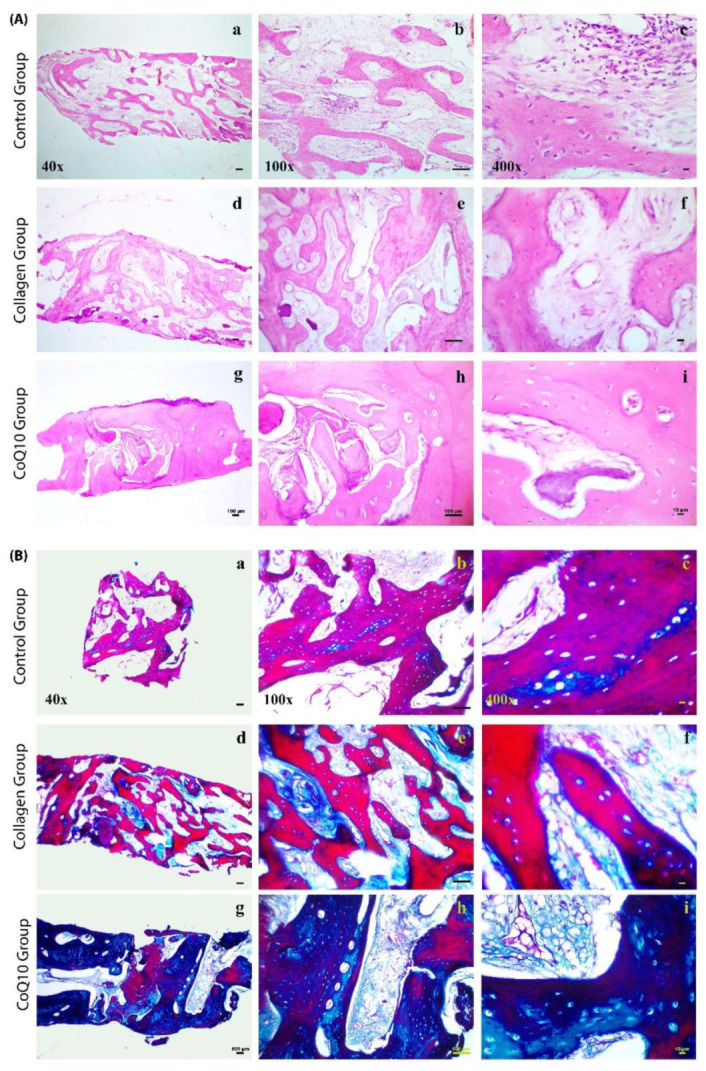
Overview of histological staining of bone core biopsies harvested at three months after healing of the extraction sockets in the three different groups. (**A**) Photomicrographs of hematoxylin and eosin-stained (H&E) sections of core biopsy samples from control ungrafted sockets, collagen group, and CoQ10 group after three months; (**a**) thin new spicules of immature woven bone were detected in various regions in the biopsy samples (40×, scale bar = 100 μm); (**b**) bone marrow loose mesenchymal tissue with reduced cellularity and vascularity (100×, scale bar = 100 μm); and (**c**) widened numerous osteocytes lacunae and few inflammatory cells were observed (400×, scale bar = 10 μm). (**d**) In the collagen group, thin segments of trabecular and woven bone (40×, scale bar = 100 μm); (**e**) fibrous tissue within the marrow cavities (100×, scale bar = 100 μm); and (**f**) Numerous wide osteocytes lacunae (400×, scale bar = 10 μm). (**g**) In the CoQ10 group, dense mature lamellar compact bone with primary and secondary osteons was revealed fused to the old bone (40×, scale bar = 100 μm); (**h**) typical trabecular bone structures were observed with clearly distinguishable osteocytes and evident resting and reversal lines (100×, scale bar = 100 μm); and (**i**) normal sized marrow cavities with mild fibrosis were also presented lined by flat endosteal cells (400×, scale bar = 10 μm). (**B**) Photomicrographs of GomoriTrichrome stained sections of core biopsy samples from control ungrafted sockets, collagen group, and CoQ10 group after three months showing the immature bone and osteoid distributions (red-stained areas) and the areas of mature bone (blue-stained areas) in the different groups: control group (**a**–**c**); collagen group (**d**–**f**); and CoQ10 group (**g**–**i**) at different magnifications (40×, 100× and 400×) three months after extraction of teeth. Scale bar = 100 μm, 100 μm and 10 μm, respectively.

On the basis of the histological specimens, it was determined that the biopsies of the grafted sockets have a greater tissue density than the biopsies of the controls. The pattern of socket healing after extraction in different groups at three months using H&E and trichrome were illustrated in Figure 7.

### 4.6. Histomorphometric Outcomes 

Histomorphometric analysis of all core biopsies in the three different groups at three months after healing of the extraction sockets were shown in Table 3. The results are expressed as Mean ± Standard deviation, 95% CI and Median (IQR) for the percentage of the bone formed and fibrous tissue at 100× magnification from the H&E sections. Only fibrous tissue and the bone formed within the extraction socket were considered, while older original bone was excluded. Histomorphometric assessments revealed a statistically significant difference between the groups in terms of percentage of formed bone (Control = 34.56 ± 8.34, Collagen = 46.00 ± 9.70, CoQ10 = 68.93 ± 9.38, *p* = 0.001). The bone formed in the CoQ10 group was statistically higher in the comparison to the collagen (*p =* 0.037) or control group (*p =* 0.001). On the contrary, CoQ10 samples exhibited a statistically significant lower percentage of fibrous tissue compared to the other two groups (*p =* 0.009). The fibrous tissue was significantly higher in the control group compared to the CoQ10 group (*p =* 0.008).

## 5. Discussion

Extraction of teeth is common in Oral and Maxillofacial Surgery due extensive carious lesions, periapical pathology, or fractured crown or root in case of trauma or chronic periodontitis [35]. Many medical conditions may alter the healing after dental extraction as diabetes mellitus which is a pandemic metabolic disease associated with several complications, such as decreased bone remodeling and lowered levels of circulating biochemical bone markers, as well as reduction in the bone density [36,37]. Patients with uncontrolled diabetes, high blood pressure, and unhealthy habits are more prone to post-surgical complications after teeth extraction [38].

As a consequence of diabetes, the soft tissue repair is affected, resulting in reduced formation of granulation tissue in addition to collagen degradation. On the bone level, it inhibits new bone formation affecting the osteoblastic function and matrix mineralization together with exacerbation of alveolar bone resorption [39,40].

Management of diabetic patients is often challenging for the Oral and Maxillofacial surgeons due to the impaired healing of the dental sockets after tooth extraction. Although the incidence of complications as post-operative infections and delayed healing is minimal in optimally controlled diabetic patients, there would still be some risk [4]. Thus, in this study, controlled diabetic patients have been chosen to be treated in a way to permit the chance for further rehabilitation by means of removable or fixed restorations.

After tooth extraction, the alveolar bone remodeling is unavoidable. The alveolar ridge undergoes vertical and horizontal bone resorption which is very rapid in the first 3 months, even though extraction is carried out following atraumatic procedures [41]. This is particularly important for implant placement as, sometimes, it is not possible to place implants immediately after tooth extraction, and, therefore, the treatment procedure should prevent alveolar ridge atrophy and maintain adequate dimensions of bone along with alveolar ridge preservation [42]. To maintain the dimension of the ridge following tooth extraction, socket grafting, also known as alveolar ridge preservation, had been commonly used to prevent bone resorption [43].

Nowadays, the increased demand for preservation of the alveolar ridge and socket grafting has created a big market for the application of biomaterials to promote soft and hard tissue healing [3]. Biomaterials have been used since ancient times, but recently their degree of sophistication has increased significantly and might incorporate biologically active components derived from nature [44]. Several grafting materials, such as platelet rich fibrin, collagen cones, hydroxyapatites, enamel matrix derivatives, and bio-glass, have also been introduced to prevent the collapse of hard and soft tissues surrounding the alveolar socket and to stabilize the blood clot [3]. 

Coenzyme Q10 (CoQ10) is among the most widely used dietary and nutritional supplements on the market. Although similar in structure to vitamin K, CoQ10 is not a vitamin since it is naturally synthesized in every cell in the human body, whereas vitamins must be obtained from the diet [45,46]. Some studies have investigated the effects of CoQ10 in diabetes being a safe material with very low toxicity [47,48,49] while other studies have shown that oral application of CoQ10 is effective in improving periodontitis [50,51,52,53]. However, the local effect of CoQ10 in bone regeneration and healing of extraction sockets in humans have not been previously studied. 

The present randomized, controlled clinical trial used CoQ10/Collagen hydrogel as a graft material for socket augmentation in Type II diabetic patients. Although collagen has been extensively used, no studies have evaluated the combination of collagen with CoQ10 in bone regeneration acting as a carrier for CoQ10 keeping it within the extraction socket. This combination was safe as CoQ10 has been commonly used as a food supplement in cardiovascular diseases or diabetes mellitus [12]. The addition of collagen favored the placement and the manipulation of the material.

From our results, the hydrogel has been formed at room temperature (25 °C) while the hydrogels were reduced when exposed to 37 °C. This should be attributed to the fact that poloxamer 407 is a thermoresponsive hydrogel. At high temperature (37 °C), the sol-gel transition from aqueous solution occurs [54]. Poloxamer 407 exists as monomers in the solution. Upon warming, equilibrium between monomers and micelles occurs, and, at higher temperatures, aggregates are formed causing increase in viscosity and gel formation. The use of poloxamer 407 as a gelling agent prolonged the residence time of the CoQ10 at the injection site, sustains drug release thus increased its therapeutic efficacy [55].

In our study, the pain intensity has decreased in the three groups from day 3 to day 7 with significant reduction in the CoQ10 group compared to the control or collagen group (*p* ≤ 0.05). This might be attributed to the anti-inflammatory effects of CoQ10 and pain reduction as reported in other studies [56,57] as CoQ10 is an antioxidant having a direct anti-inflammatory properties by suppressing TNF-α gene expression in mice and exerts anti-inflammatory effects probably via NF-κB1-dependent gene expression [58].

The results of the present study observed that implant stability gradually decreased three months after placement. There was no significant difference between the primary implant stability between the three different groups. However, there was decrease in the secondary stability between the groups, but the reduction was not statistically significant. Despite this decrease, the implants remained clinically stable throughout the whole period. This might be attributed to the implant stability dip period [59]. By definition, implant stability is the absence of clinical implant mobility and classified as primary and secondary implant stability where the primary stability is gained mechanically during the implant insertion; and the secondary stability is gained biologically after healing and is the result of osseointegration [60]. 

Although very high primary stability is regarded as beneficial, it does not necessarily entail greater secondary stability. It might be related to the inflammatory process subsequent to implant installation with resorption of the bone tissue immediately lateral to the pitch region in discrete areas which is responsible for primary mechanical stability of the implant and replaced with newly formed viable bone [61].

The bone density was measured in this study by CBCT providing a measure of the extent of bone mineralization. As the new bone maturates, more mineralization is detected [62]. In this study, bone mineral density was significantly higher in the CoQ10 group compared to sockets treated with collagen alone or where the sockets were left ungrafted (control group). Therefore, the bone mineral density measure between control, collagen, and CoQ10 groups reflects the advanced healing and maturity of vital bone in the CoQ10 treated sockets.

After tooth extraction, consequent bone remodeling started, and post-extraction bone loss was significantly the highest on the buccal aspect of the alveolar process. This may be attributed to the higher proportion of resorption-prone bundle bone of the buccal plate because it is generally thinner [63]. Previous studies were in agreement to our results and demonstrated a greater amount of bone loss on the buccal plate of bone when compared to the lingual side [64,65,66] as bone resorption occurs in two phases. During the first phase, bundle bone is rapidly resorbed and replaced with woven bone leading to a great reduction in bone height, especially in the buccal aspect of the socket, as its crestal portion is comprised solely of bundle bone [67].

CBCT scan assessments showed that there was no statistically significant difference between the groups in terms of the height of the lingual plate of bone, and the width and volume of the extraction socket. The magnitude of the effect of socket preservation therapies on linear bone reduction is consistent with the information reported in the majority of studies about this topic [68,69]. From our results, there was no significant difference in the width of the socket at 1, 3, and 5 mm, indicating that the width of the socket was preserved, but what made the difference is using CoQ10 that had affected the quantity and quality of the bone formed inside the socket which is important in the bone regeneration and subsequent implant placement to ensure success of the dental implant. Notably, no site required additional bone augmentation prior to or at the time of implant placement in this study. The socket augmentation has preserved the width of the socket while the use of COQ10 has improved the bone formation and maturation at the same timepoint. From our findings, the socket preservation by insertion of collagen or CoQ10/Collagen to the tooth extraction socket has the advantage of promoting bone healing in the extraction socket and is better than leaving the extraction socket filled with nothing but the natural blood clot. A previous study reported that CoQ10 enhances bone-forming osteoblast differentiation and suppresses osteoclast differentiation compared to ungrafted extraction sockets [19].

We have to report that it is not common to use PCR techniques in the detection of bone formation in the extraction socket. The most commonly used technique is the histology and histomorphometry. However, we have performed RT-qPCR for the bone markers expressed in the extraction socket. From our results, there were significant increases in the OPN and RUNX2, while OCN and COL1A1 were not significantly increased. This could be explained by RUNX2 being a marker of an early-stage bone cell differentiation and the expression of OPN is mainly associated with bone metabolism and remodeling and relatively late-stage osteogenic differentiation [70]. COL1A1 is the main component of extracellular matrix and is also one of the marker genes expressed in early stage of osteogenic differentiation [71]. No significant differences were detected among the three groups for the OCN values at 3 months after healing of the extraction sockets that might be due to the short-term study period (3 months) that did not allow sufficient bone formation to occur, which, in term, affects the osteocalcin levels [72]. OPN expression showed higher values in the CoQ10 group than in the other groups at three months of healing of the extraction sockets that was in an agreement to a study showing that CoQ10 protects against osteoporosis and may regulate bone metabolism indicating CoQ10 as a potential safe therapeutic used to treat human diseases [73]. 

The histological results of the core bone biopsy samples demonstrated significant regeneration of mature bone in the CoQ10 sockets, demonstrating bone lamellae, osteocytes, Haversian systems, and osteoblasts. Bone maturation was confirmed by the homogeneous distribution of healthy osteocytes, as well as the organized Haversian canals. The trichrome staining also provided evidence for the maturation of bone. In conjunction with bone regeneration, complete resorption of the graft material was observed three months after extraction. The newly formed bone provided successful and sustained volumetric and implant stability. Histomorphometry demonstrated a statistically significant higher quantity of new vital bone in the CoQ10 group than was formed in the control ungrafted sockets or the collagen, which demonstrated immature woven bone and higher amount of fibrous tissue formation. The hypothesis that the application of the socket graft material would lead to a reduction in bone degeneration after tooth extraction was therefore confirmed. 

The use of collagen showed mild or no enhancement of the bone healing outcome after extraction compared to leaving the extraction socket ungrafted. One explanation for that outcome was that the augmentation materials might interfere with vascularization supply of the soft tissue from underlying bone. There was no advantage in terms of dimensional changes in alveolar bone and soft tissue after tooth extraction in alveoli grafted with collagen compared with those left ungrafted.

Following tooth extraction, blood clotting is followed by vascular granulation tissue formation, mineralization, and bone remodeling to finally yield a structured hard tissue [74]. In terms of histological results, several studies reported significantly higher amount of new bone formation and difference in the trabecular bone structure in socket augmentation than that in ungrafted socket healing after a 3-month healing period [75,76].

The present study has some limitations including the small sample size in total and per group and short period of follow-up (3 months). However, the results of this randomized controlled clinical trial provided valuable information on the use of Coenzyme Q10 for the management of extraction sockets, especially in medically compromised patients that have conditions affecting the soft tissue healing and bone formation.

## 6. Conclusions

A potential complication of tooth extraction is a socket exposed to the oral environment leaving an open wound which may serve as a portal for serious pathogens. Innovations in Oral and Maxillofacial Surgery have stepped towards socket preservation and regeneration. Based on our study findings, the local application of CoQ10 within the extraction socket provided a simple, inexpensive, yet effective technique in healing of extraction socket and bone formation especially in diabetic patients. In addition, CoQ10 may act as a routine procedure for post-extraction socket care and preservation of the alveolar bone prior to implant placement. It might also be advantageous to test CoQ10 in large bone defects to show the impact on bone regeneration. 

## Figures and Tables

**Table 1:** Implant Stability.

Variable	Control	Collagen	CoQ10	*p*-Value
**At Insertion**				
Mean ± SD	61.83 ± 4.91	56.60 ± 7.58	61.13 ± 8.29	0.168
95% CI	49.64, 74.02	47.19, 66.01	47.93, 74,32	
Median (IQR)	59.00	55.00	57.50	
**At three Months**	52.2 ± 0.74	53.8 ± 2.17	56.6 ± 2.39	
Mean ± SD	52.17 ± 5.97	53.80 ± 6.75	56.63 ± 7.75	0.304
95% CI	37.35, 66.98	45.42, 62.18	44.30, 68.95	
Median	49.50	50.25	53.63	

**Table 2:** Radiographic Outcomes of CBCT at baseline and three months later.

Variable	Control	Collagen	CoQ10	*p*-Value
**Bone Density (Mean Grey Value)**				
Mean ± SD	6.33 ± 1.17	6.52 ± 1.91	18.93 ± 7.28	0.003 *
95% CI	5.10, 7.56	4.52, 8.52	11.28, 26.57	
Median (IQR)	5.84 (2.24)	6.56 (3.32)	17.23 (13.26)	
**Percentage of Bone formation in the extraction socket**				
Mean ± SD	7.52 ± 1.58	7.73 ± 2.38	22.38 ± 7.85	0.003 *
95% CI	5.86, 9.17	5.24, 10.23	14.14, 30.61	
Median (IQR)	7.20 (3.08)	7.51 (4.33)	19.3 (13.15)	
**Difference in height of buccal plate of bone (mm)**				
Mean ± SD	2.54 ± 0.13	1.99 ± 0.90	0.92 ± 0.69	0.011 *
95% CI	2.17, 2.92	1.04, 2.94	0.19, 1.65	
Median (IQR)	2.62 (0.63)	1.86 (1.67)	0.82 (1.27)	
**Difference in height of lingual plate of bone (mm)**				
Mean ± SD	1.24 ± 0.21	1.41 ± 0.51	1.09 ± 0.38	0.493
95% CI	1.03, 1.46	0.88, 1.95	0.69, 1.49	
Median (IQR)	1.30 (0.42)	1.39 (0.97)	1.00 (0.78)	
**Difference in width of socket at 1 mm from alveolar crest** **(mm)**				
Mean ± SD	1.36 ± 0.52	1.31 ± 0.58	1.07 ± 0.58	0.700
95% CI	0.82, 1.91	0.70, 1.92	0.46, 1.68	
Median (IQR)	1.36 (1.05)	1.30 (0.81)	1.07 (1.02)	
**Difference in width of socket at 3 mm from alveolar crest (mm)**				
Mean ± SD	1.22 ± 0.28	1.14 ± 0.73	0.93 ± 0.44	0.644
95% CI	0.93, 1.51	0.37, 1.91	0.48, 1.39	
Median (IQR)	1.20 (0.49)	0.98 (1.48)	0.87 (0.92)	
**Difference in width of socket at 5 mm from alveolar crest (mm)**				
Mean ± SD	0.87 ± 0.35	0.73 ± 0.81	0.47 ± 0.29	0.262
95% CI	0.50, 1.24	0.12, 1.58	0.17, 0.77	
Median (IQR)	0.86 (0.73)	0.43 (1.15)	0.40 (0.40)	
**Volume of the extraction socket immediate after extraction**				
Mean ± SD	79.36 ± 46.42	106.76 ± 63.90	115.69 ± 66.28	0.548
95% CI	30.64, 128.07	39.71, 173.82	46.14, 185.25	
Median (IQR)	72.43 (58.88)	93.73 (125.96)	99.67 (133.82)	
**Volume of the extraction socket at three months**				
Mean ± SD	43.03 ± 20.16	69.53 ± 37.33	54.47 ± 25.39	0.348
95% CI	21.88, 64.19	30.36, 108.70	27.82, 81.12	
Median (IQR)	37.22 (37.70)	68.13 (58.10)	48.70 (38.26)	

* *p* ≤ 0.05.

**Table 3:** Histomorphometric analysis of the tissue formed at three months after healing of extraction socket.

Variable	Control	Collagen	CoQ10	*p*-Value
**Percentage of bone formed**				
Mean ± SD	34.56 ± 8.34	46.00 ± 9.70	68.93 ± 9.38	0.001 *
95% CI	26.84, 42.27	37.02, 54.97	60.26, 77.60	
Median (IQR)	33.47 (9.36)	48.98 (12.10)	65.11 (11.89)	
**Percentage of fibrous tissue formed**				
Mean ± SD	34.89 ± 3.07	27.24 ± 6.86	12.96 ± 1.21	0.009 *
95% CI	30.01, 39.78	20.89, 33.59	11.03, 14.88	
Median (IQR)	33.83 (5.0)	24.73 (13.0)	13.05 (2.0)	

* *p* ≤ 0.05.

## Data Availability

Not applicable.

## Appendix A

**Table A1:** Mankowski Pain Scale.

Mankowski Pain Scale		
0	Pain Free	No medication needed.
1	Very minor annoyance—occasional minor twinges.	No medication needed.
2	Minor annoyance—occasional strong twinges.	No medication needed.
3	Annoying enough to be distracting.	Mild painkillers are effective. (Aspirin, Ibuprofen.)
4	Can be ignored if you are really involved in your work, but still distracting.	Mild painkillers relieve pain for 3–4 h.
5	Can’t be ignored for more than 30 min.	Mild painkillers reduce pain for 3–4 h.
6	Can’t be ignored for any length of time, but you can still go to work and participate in social activities.	Stronger painkillers (Codeine, Vicodin) reduce pain for 3–4 h.
7	Makes it difficult to concentrate, interferes with sleep You can still function with effort.	Stronger painkillers are only partially effective. Strongest painkillers relieve pain (Oxycontin, Morphine)
8	Physical activity severely limited. You can read and converse with effort. Nausea and dizziness set in as factors of pain.	Stronger painkillers are minimally effective. Strongest painkillers reduce pain for 3–4 h.
9	Unable to speak. Crying out or moaning uncontrollably—near delirium.	Strongest painkillers are only partially effective.
10	Unconscious. Pain makes you pass out.	Strongest painkillers are only partially effective.
